# W/Mo/Cr Doping Modulates the Negative–Positive Inversion Gas Sensing Behavior of VO_2_(M1)

**DOI:** 10.1021/acssensors.4c03006

**Published:** 2025-01-09

**Authors:** Lei Miao, Yibei Xue, Peng Song, Takuya Hasegawa, Ayahisa Okawa, Ryo Maezono, Tohru Sekino, Shu Yin

**Affiliations:** † Institute of Multidisciplinary Research for Advanced Materials (IMRAM), Tohoku University, Sendai 980-8577, Japan; ‡ School of Information Science, JAIST, Asahidai 1-1, Nomi, Ishikawa 923-1292, Japan; § SANKEN, Osaka University, Osaka 567-0047, Japan; ∥ Advanced Institute for Materials Research (WPI-AIMR), Tohoku University, Sendai 980-8577, Japan

**Keywords:** doping, modulation, gas sensing behavior, inversion, negative−positive, VO_2_(M1)

## Abstract

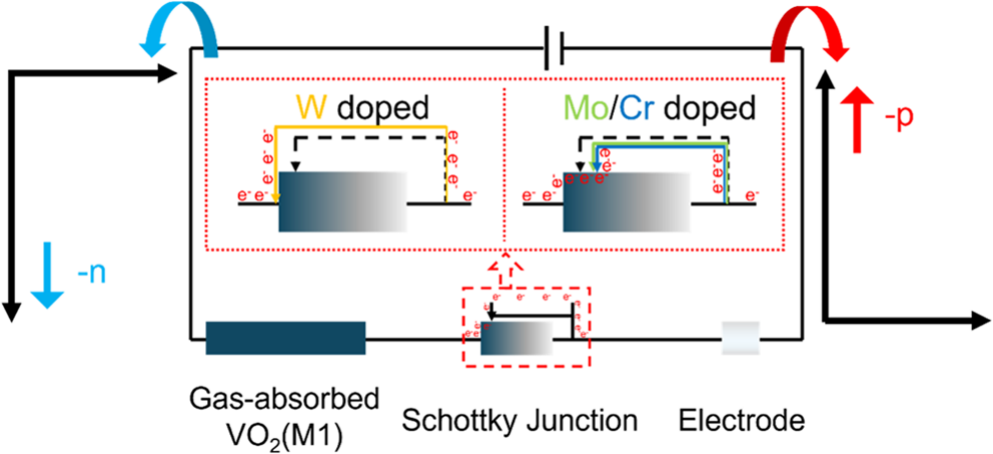

The anomalous gas sensing behavior has garnered significant attention from researchers, prompting a re-evaluation of the gas sensing theory. This work focuses on inversion gas sensing behavior induced by element doping. W/Mo/Cr-doped VO_2_(M1) samples are synthesized, and their sensing behaviors are investigated. The results show that the elements can modulate the sensing behavior with an opposite orientation. The sensing behavior in the opposite orientation is attributed to the extent of the reduced Fermi level of VO_2_(M1) after doping. W-doped VO_2_(M1) maintains a resistance-decreased sensing behavior (-n). In contrast, the decrease in Fermi level results in the formation of a Schottky barrier between the gas-absorbed Mo/Cr-doped VO_2_(M1) and the electrode. The formation of Schottky barriers leads to the inversion sensing behavior, which feedbacks as an increased resistance (-p). This study offers a novel perspective on the gas sensing theory.

The contribution of the gas sensor to health monitoring, environmental assessment, and industrial process control is undeniable.^1−5^ Gas sensors are composed of sensing materials, electrodes, and the associated processes. It provides critical feedback on the status of target gases in the environment by monitoring their responses. As indicators for evaluating the gas sensing material, response and selectivity have become the factors for establishing a correspondence relationship between the sensing material and gas.^6−9^ Response is defined as the degree of signal difference observed before and after gas adsorption. Meanwhile, selectivity is the significant difference in the signal between the target gas and other gases. Typically, selectivity is known as a significant difference in response.^6,7^ Even though it is not defined, such selectivity has been widely used since the discovery of a metal oxide semiconductor (MOS) sensing material. However, relying solely on the response value for selectivity presents a significant drawback. When a mixed gas is tested for sensing, the responses of other gases may interfere with the response of the target gas. This is obviously a major problem in the application of the MOS gas sensor.

In recent years, the report of selectivity that is evaluated by the sensing behavior change seems to be shedding more light on the solution to the above problem.^200^ Sensing behavior change, which is induced by the operation temperature, has been used as an indication of gas selectivity. Such a behavior change is referred to as p–n,^10−17^ n–p,^18−20^ n–p–n,^21^ and p–n–p^22^ transitions. These transitions are all defined as the change in the semiconductor type or the method of charge transfer in the sensing material itself by doping or compositing. Naturally, the semiconductor type cannot be easily changed. Therefore, it is important to understand the reason for the sensing behavior changes. The studies mentioned above mainly reported the inversion sensing behavior at certain temperatures. However, the other factors responsible for the inversion sensing behavior are still unknown. On the other hand, the temperature-induced inversion behavior has been attributed to the differences in the oxygen absorption form (change in the oxygen absorption form from O^–^ to O^2–^ on heating).^8^ Once the inversion behavior occurs at the same temperature by other factors, the reason for its occurrence cannot be well explained by the change in the oxygen absorption form. Therefore, discovering the sensing behavior change at the same temperature will potentially advance the development of sensing theory.

Vanadium dioxide (VO_2_) is well known for its rich crystal structure and excellent semiconducting properties.^23,24^ Among these structures, the monoclinic structure (VO_2_(M1)) can undergo a reversible metal–semiconductor phase transition (MST) with VO_2_(R) at near-room temperature (about 68 °C).^25,26^ This means that VO_2_(M1) exhibits semiconducting characteristics at room temperature. In our previous work, VO_2_(M1) was studied to develop its infrared shielding applications. Elemental doping can effectively lower its MST temperature while simultaneously enhancing infrared shielding.^27−30^ In addition, VO_2_(M1) is a typical n-type gas sensing material.^23,31−34^ Therefore, it is possible to improve the sensing characteristics by decreasing the MST temperature of VO_2_(M1). This means that VO_2_(M1) has the potential to improve its sensing characteristics by doping and even to inverse and modulate its sensing behavior.

Hence, the dopant elements are initially screened by density functional theory (DFT) calculations in this study. Based on these results, VO_2_(M1) is doped with W, Mo, and Cr. The relationship between the dopant amount and MST temperature is established. The gas sensing characteristics of 0.5 at. % W/Mo/Cr-doped VO_2_(M1) are investigated. The inversion sensing behavior due to elemental doping is further verified as well as the other factors for influencing the sensing behavior are summarized. This study also provides an experimental foundation for understanding gas sensing mechanisms.

## Experimental Section

### Computational Details for Screening Doped Element

To further explore the feasibility of doping in VO_2_(M1), this study employs density functional theory (DFT) to calculate the formation energy of defects following the doping of additional elements. A 2 × 2 × 2 supercell model comprising 96 atoms is constructed; one vanadium atom is replaced with a metal element *X*, where *X* represents one of the following doping elements: Sc, Ti, Cr, Mn, Fe, Zr, Nb, Mo, Hf, Ta, or W. The doped structure is designated as V_31_XO_64_, which helps to assess its potential impact on material properties.

For the total energy calculations of these doped structures, we utilize the generalized gradient approximation (GGA) Perdew–Burke–Ernzerhof (PBE) functional,^35^ combined with the projected augmented wave (PAW) method,^36^ all performed in the Vienna *Ab-initio* Simulation Package (VASP).^37−40^ The simulations use a 4 × 4 × 4 *K*-point mesh and 600 eV cutoff energy. During structural optimization, antiferromagnetic (AFM) spin configurations are considered, and the Hubbard model (DFT+*U*)^41^ is applied to correct the ***d*** orbitals of vanadium. The *U* value for vanadium is set at 2.7 eV based on the AFLOW library,^42^ with the corresponding adjustments made for the *U* values of the doped elements *X* based on their respective data from AFLOW.

The defect formation energy, ***E***^*f*^[*X*^*q*^], is calculated by comparing the energy of the doped system obtained from simulations with known data from the Material Project database^43^ to assess the doping’s stability and feasibility. The formula for the defect formation energy is as follows
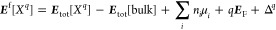
1where ***E***_tot_[*X*^*q*^] represents the total energy of the system containing the doping element *X* with charge *q*. ***E***_tot_[bulk] is the total energy of the defect-free system. ∑_*i*_*n*_*i*_μ_*i*_ is the sum of the product of the chemical potential μ_*i*_ and the number *n*_*i*_ of the defect component element *i*. *q***E**_F_ is the product of charge *q* and Fermi energy ***E***_F_. Δ^*q*^ is a correction term for the charged state *q*.

Chemical potential μ_*i*_ is calculated based on μ_0_ (the standard energy of the reference phase) and Δμ_*i*_ (the deviation from the standard energy) (Table S1). In the V–O–X ternary system, Δμ values are obtained using Pymatgen software^44^ to determine the competing phases. Additionally, the correction scheme proposed by Freysoldt et al.^45^ is also employed to address the electrostatic interaction between the periodic images of the charges and the band shift caused by the introduction of the defect. All defect calculations are set up and analyzed using the Pymatgen-analysis-defects module,^46^ ensuring accuracy and efficiency in our computations.

### Synthesis of the W/Mo/Cr-Doped VO_2_(M1)

All reagents used in this study are of analytical grade and used without further purification.

3.33 cm^3^ s^–1^ (200 sccm) of N_2_ (99.9995%, Taiyo Nippon Sanso Co.) is passed into a beaker, including 1.00 g of vanadium pentoxide (V_2_O_5_, >99.0%, Kanto Chemical Co. Inc.) and 28 mL of water. Meanwhile, ammonium tungstate para pentahydrate (Fujifilm Wako Pure Chemical Co. Ltd.), ammonium molybdate (Fujifilm Wako Pure Chemical Co. Ltd.), and chromium oxide (Kanto Chemical Co. Inc.) are added as the corresponding dopant sources in the desired doping amounts. After mixing well, 5 mL of 30% aqueous hydrogen peroxide solution (30.0–35.5%, Kanto Chemical Co. Inc.) is added to the beaker and completely dissolved by stirring (600 r min^–1^) at 60 °C for 24 h to form a dark reddish-brown gel. 0.133 mL of hydrazine monohydrate (98%, Fujifilm Wako Pure Chemical Co. Ltd.) was subsequently added into the gel and manually stirred (20 min) to completely transform the gel into a dark blue-green gel of the V precursor. To construct the relationship between the dopant amount and the MST temperature, the tungsten source is added as 0.5, 1.0, 1.5, 2.0, 2.5, 3.0, and 5.0 at. % to form W-VO_2_(M1) (the corresponding samples after the supercritical fluid reaction are named W0.5, W1.0, W1.5, W2.0, W2.5, W3.0, and W5.0, respectively). Similarly, the molybdenum source is added as 0.5, 1.0, 1.5, 2.0, 2.5, 3.0, and 5.0 at. % to form Mo-VO_2_(M1) (the corresponding samples after the supercritical fluid reaction are named Mo0.5, Mo1.0, Mo1.5, Mo2.0, Mo2.5, Mo3.0 and Mo5.0, respectively), and the chromium source is added as 0.2, 0.4, 0.5, 1.0, and 1.5 at. % to form Cr-VO_2_(M1) (the corresponding samples after the supercritical fluid reaction are named Cr0.2, Cr0.4, Cr0.5, Cr1.0, and Cr1.5, respectively).

The supercritical fluid reaction is performed in a Hastelloy tube with a volume of 20 mL. 200 sccm of N_2_ (200 sccm) is first filled into the Hastelloy tube for 2 min. Then, 5 g of the V precursor and 10 mL of water are added to the Hastelloy tube separately while keeping the N_2_ flux. After the N_2_ was passed into the Hastelloy tube for 1 min, the Hastelloy tube was fully assembled and subjected to the supercritical fluid reaction at 490 °C for 30 min. The synthesized powder was centrifuged at 15,000 r min^–1^ for 20 min at 18 °C and then filtered and washed with deionized water and ethanol alternately three times. The powder is then dried under vacuum at 60 °C for 12 h.

### Gas Sensing Characteristic Investigation

Ethanol (0.2 mL) is added into a container with 10 mg of as-synthesized powder and dispersed under ultrasonic conditions for 2 min to make the bottom of the container free of precipitate. The above solution is dispersed as slurry on a Ag interdigitated electrode with a ceramic substrate at 60 °C, and 0.015 mL d^–1^ of the slurry is dropped onto the silver interdigitated electrode for 4 drops. The as-fabricated sensor is then placed in a vacuum oven at 60 °C overnight.

The as-fabricated sensor is connected to the gas sensing system consisting of gas and gas paths, a flow meter (SEC-N112MGMW, HORIBA), a gas chamber, a gas room, a heater, input and output thermocouples, an impedance analyzer unit of 34970A (Agilent), and an exhaust discharge and treatment unit. The calibrated standard NH_3_ (24.7 ppm), H_2_S (5.00 ppm), H_2_ (0.97 ppm), and NO (2.93 ppm), which are purchased from Taiyo Nippon Sanso Co., are used as the target gases, while N_2_ (99.99995%, H_2_O < 0.54 ppm while the humidity is 0.00% RH when the temperature is 20 °C, Taiyo Nippon Sanso Co.) is used as the base gas. When the gas sensing characteristics are investigated, the flow rate of the gas in the sensing system is always controlled at 200 sccm. The gas sensing testing is carried out at different target gas concentrations and desired operation temperatures. The target gas concentrations are regulated by the flow meter to 10, 20, 30, 40, 50, and 100% of the calibrated target gas concentrations, respectively. The operation temperatures (ambient temperature) are 20, 30, 40, 50, and 60 °C (error: ±0.3 °C), which set the heating unit controlled by input thermocouples and output thermocouples, indicating the actual ambient temperatures, respectively.

The sensing response (*R*) is evaluated by the following equations
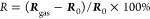
2where ***R***_0_ is the resistance of the gas sensing when the gas is in. And ***R***_gas_ is the resistance of the gas sensor when the gas is in and reaches a plateau value.

### Physical Characterization

The phase information on the samples is recorded by an X-ray powder diffractometer (Bruker D2 Phaser diffractometer, Bruker, Cu Kα). The MST behavior of the sample is monitored by a differential scanning calorimetry (DSC, DSC vesta, Rigaku) at a heating or cooling rate of 5 °C min^–1^ in a N_2_ atmosphere. X-ray photoelectron spectroscopy (XPS) is acquired from a PHI5600 (ULVAC PHI Co., Ltd.). Argon ion etching is performed for 3 min before measurements to eliminate the interference of contaminants on the results. The transmission electron microscopy (TEM) images of the sample morphology are obtained by TEM (JEM-2100F, JEOL) operated at 200 kV. The specific surface area of W0.5, Mo0.5, and Cr0.5 is tested by the surface area and pore size analyzer (QUADRA SORB EVO4, Quantachrome Instruments). The average of three measurements for each sample is taken as their respective specific surface area.

## Results and Discussion

### Screening and Synthesis of W/Mo/Cr-Doped VO_2_(M1)

**Figure 1:**
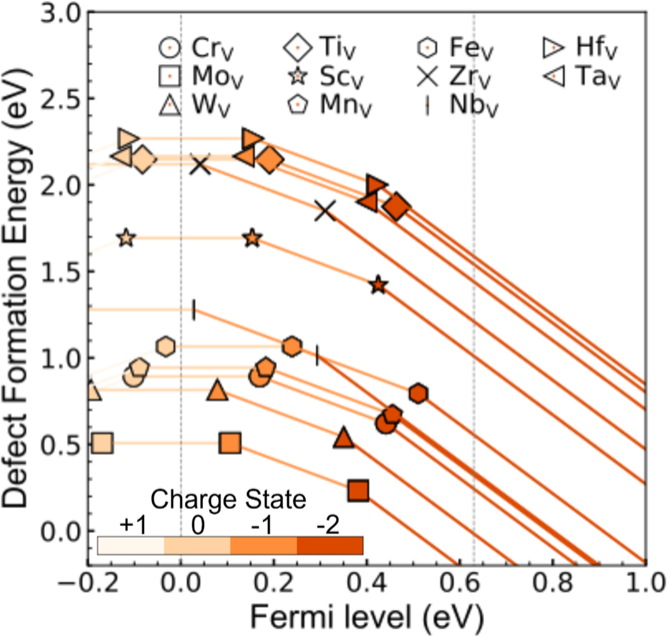
Defect formation energy as a function of the Fermi level in VO_2_(M1) with an approximate band gap of 0.63. The analysis exclusively addresses extrinsic defects induced by the substitution of 11 different elements (*X*) at the vanadium site (*X*_V_). The energy states of the positively charged electron-donor and negatively charged electron-acceptor defects are indicated by different colors, which are shown in the color bar.

The relationship between the Fermi level and defect formation energy in VO_2_(M1) is initially investigated, evaluating different defects in their respective charge states across competing phases (Figure 1). The defect formation energies are influenced by the magnitude of the band gap and the energies of individual band edges, with the gradient of each line reflecting the charge states of the defects. The Fermi level at which the formation energy of a defect *X* in two charge states becomes equal is termed the thermodynamic transition level. VO_2_(M1) is an n-type semiconductor with a band gap of approximately 0.63 eV. In n-type semiconductors, the formation energies of these dopants range from 0.0 to 2.5 eV. Regardless of how the Fermi level shifts, either as an electron acceptor or donor, doping with group VI elements such as W, Mo, and Cr results in the lowest defect formation energies. Additionally, doping with W, Mo, and Cr effectively tunes the Fermi level and the band structure.^47−51^ Based on the Mott–Schottky model, the Schottky barrier height should be^52−54^
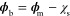
3where ***ϕ***_b_ is the Schottky barrier height. ***ϕ***_m_ is the barrier height of the electrode. And χ_s_ is the electron affinity of the semiconductor, defined as the difference in energy between an electron at rest outside the surface and an electron at the bottom of the conduction band just inside the surface.

Due to the reduction of the Fermi level, it is possible that the conduction band of VO_2_(M1) will decrease. Therefore, χ_*s*_ is changed and further causes the doped VO_2_(M1) to change in its Schottky barrier. The higher Schottky barrier may cause a current in the opposite orientation, which can be explained by the current density (***J***) in the thermionic emission theory^54,55^
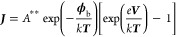
4where A** is the effective Richardson constant for thermionic emission, neglecting the effects of optical phonon scattering and quantum mechanical reflection (equal to 120 A cm^–2^ K^–2^ for free electrons), ***T*** is the absolute temperature, *k* is the Boltzmann constant, and ***V*** is the bias voltage.

The thermionic emission theory further means that the decrease in the Fermi level favors the increase in electron affinity. Therefore, the decrease in the Fermi level may increase the Schottky barrier height. The formed Schottky barrier can generate a higher reverse current. The reverse current is thus further likely to invert the sensing behavior of doped VO_2_(M1). Therefore, all 11 elements examined have the potential to achieve the purpose of modulating the sensing behavior. Meanwhile, it is obvious that the stability of the doped structures can be evaluated directly by the defect formation energy. Mo-, W-, and Cr-doped VO_2_(M1) become the structures with the smallest defect formation energy, whereas the Hf-, Ti-, and Ta-doped VO_2_(M1) become the structures with the largest defect formation energy. Hence, the evaluation of the element-doped VO_2_(M1) reveals that the Fermi level of VO_2_(M1) can be reduced by elemental doping. Based on the selection of the most stable structure, W, Mo, and Cr are chosen as the doping elements.

**Figure 2:**
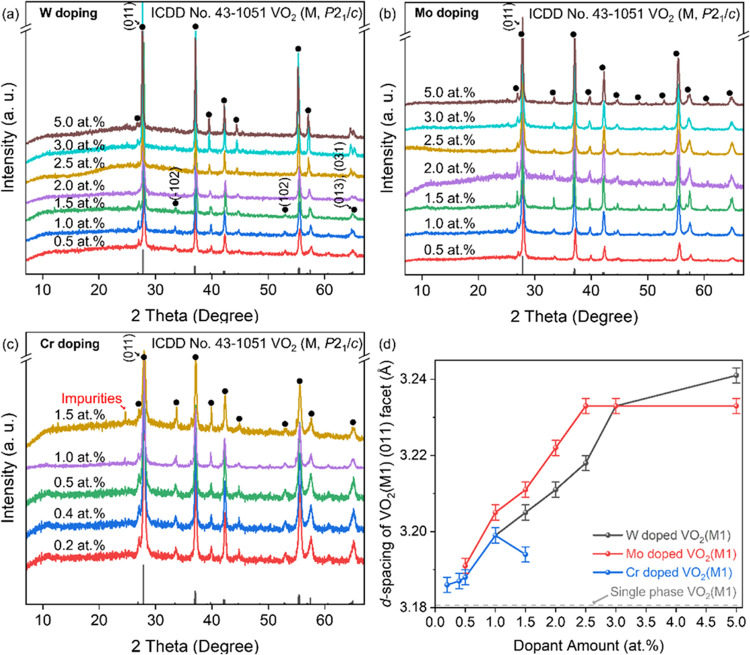
(a–c) XRD patterns and (d) the relationship between the element dopant amount and the ***d***-spacing of the diffraction peak of the VO_2_(M1) (011) facet of the (a) W-, (b) Mo-, and (c) Cr-doped VO_2_(M1) samples.

W-VO_2_(M1), Mo-VO_2_(M1), and Cr-VO_2_(M1) all result in the growth of the cell parameters of VO_2_ (M1). The XRD diffraction peaks of the doped VO_2_(M1) are left-shifted compared to that of the pristine VO_2_(M1).^27−30^ Tungsten readily doped with VO_2_(M1) for forming W-VO_2_(M1) (Figure 2a). The XRD pattern of W0.5 still exhibits the diffraction peaks characteristic of VO_2_(M1), including the (011), (−102), (102), (013), and (031) facets. However, these peaks shift to the left compared to pristine VO_2_(M1).^29^ This left shift is also observed in W1.0, W1.5, and W2.0, with the degree of shift increasing with the dopant concentration. Notably, at 2.5 at. %, the XRD pattern still displays peaks attributed to VO_2_(M1), but the peak at 2θ ≈ 65° splits, indicating the coexistence of VO_2_(R) and VO_2_(M1). It is predicted that W2.5 lowers the MST temperature of VO_2_(M1) to near-room temperature with a more pronounced left shift of the (011) peak. As the tungsten concentration is increased to 3 and 5 at. %, the peak splitting at 2θ ≈ 65° becomes more significant, further predicting a decrease in the MST temperature. The left shift of the XRD peaks also becomes more pronounced, reaching a maximum in the W5.0 sample. Mo-doped VO_2_(M1) also shows left-shift diffraction peaks (Figure 2b), with continuous shifts observed as the dopant concentration increases from 0.5 to 2.5 at. %. However, the diffraction peaks do not shift further when the concentration exceeds 2.5 at. %. It suggests that Mo doping with VO_2_(M1) is limited beyond this concentration. Additionally, no splitting at 2θ ≈ 65° is observed during Mo doping, indicating that Mo doping does not lead to the formation of VO_2_(R). The formation of Cr-doped VO_2_(M1) is more challenging (Figure 2c). Cr-VO_2_(M1) can only be synthesized at Cr concentrations of 0.5 at. % or lower (Cr0.2, Cr0.4, and Cr0.5). The XRD peaks also shift left with an increasing Cr concentration. However, the XRD pattern of Cr1.0 shows a weak impurity peak at 2θ = 24°, while Cr1.5 shows an even higher impurity peak at the same position. This indicates that Cr doping into the VO_2_(M1) structure becomes difficult when the dopant concentration exceeds 0.5 at. %. The synthesis process of the doped VO_2_(M1) demonstrates that W/Mo/Cr can effectively serve as a dopant. The diffraction peaks of the samples shift left with an increasing dopant concentration, indicating that doping enhances the cell parameters of VO_2_(M1). Specifically, 0.5–5.0 at. % W, 0.5–2.5 at. % Mo, and 0.2–0.5 at. % Cr can be successfully doped into the VO_2_(M1) structure.

To clarify the relationship between the dopant amount and cell parameters, the correlation between the dopant amount and the ***d***-spacing of the (011) diffraction peak of the doped VO_2_(M1) is presented (Figures 2d and S1, calibrated by the NaCl crystal). It is evident that the dopant amount does not maintain a linear relationship with cell parameters. However, the relationship between the dopant amount and the (011) peak position illustrates the impact of dopants on the cell parameters of VO_2_(M1). The most significant shift in the (011) peak position occurs with tungsten doping, where the ***d***-spacing increases from 3.188 Å (W0.5) to 3.241 Å (W5.0). This phenomenon can be attributed to two factors. On the one hand, W occupies the V-site in the crystal structure of VO_2_, leading to increased cell parameters. On the other hand, the presence of VO_2_(R) causes the (011) peak of VO_2_(M1) to converge with that of VO_2_(R). For Mo doping, the ***d***-spacing of the (011) facet increases from 3.191 Å (Mo0.5) to 3.233 Å (Mo2.5). Beyond 2.5 at. % Mo, the ***d***-spacing does not change, indicating that molybdenum no longer dopes into the VO_2_(M1) crystal structure at higher concentrations. Cr doping causes the least significant change in the cell parameters of VO_2_(M1), with a small shift in ***d***-spacing from 3.186 to 3.180 Å for 0.2–0.5 at. % Cr. When the dopant amount exceeds 0.5 at. %, the (011) peak position changes irregularly due to the formation of impurities.

Meanwhile, the MST behavior of W-, Mo-, and Cr-doped VO_2_(M1) is analyzed by DSC (Figure S2). As thermal hysteresis is induced by doping, the MST behavior of doped VO_2_(M1) is defined as the temperature corresponding to the endo/exothermic peaks in the DSC curve. Doping with all three elements results in a decrease in the MST temperature of VO_2_(M1) (Figure S2d). The most significant change was observed with W-VO_2_(M1). Specifically, 2.5 at. % W can reduce the MST temperature to room temperature (Figure S2a), and concentrations greater than 2.0 at. % tungsten accelerate the decrease in MST temperature, with 5.0 at. % W reducing it to below 0 °C (Figure S2a). Molybdenum also lowers the MST temperature of VO_2_(M1) but to a lesser extent than that of tungsten (Figure S2b). Chromium doping results in the smallest decrease in the MST temperature, accompanied by the most pronounced thermal hysteresis during the phase transition (Figure S2c). Importantly, the MST behavior of VO_2_(M1) remains reversible regardless of the dopant. Based on the considerations of phase composition, MST temperature, and MST behavior, W0.5, Mo0.5, and Cr0.5 are selected for further investigation of their gas sensing behavior.

### Characterization of 0.5 at. % W/Mo/Cr-VO_2_(M1)

**Figure 3:**
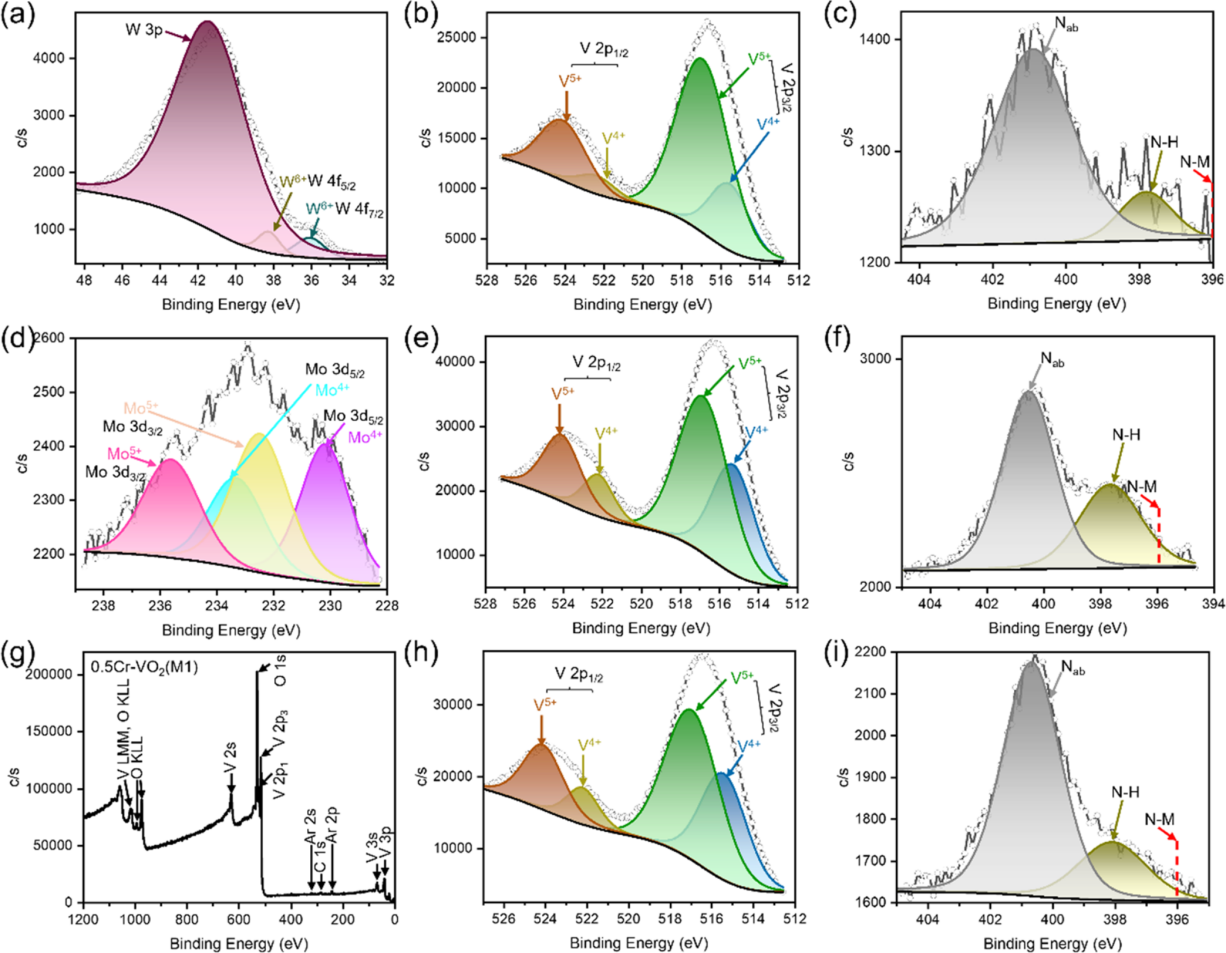
(a) W 4f, (b, e, h) V 2p, (c, f, i) N 1s, (d) Mo 3d, and (g) full spectra of (a–c) W0.5, (d–f) Mo0.5, and (g–i) Cr0.5 samples by XPS spectra.

The charge states of W0.5, Mo0.5, and Cr0.5 are further investigated to understand the status of the element after doping. The XPS spectra of W, Mo, Cr, V, and N are investigated (Figures 3 and S1). The peaks at 36.08 and 38.26 eV can be assigned to W 4f_7/2_ and W 4f_5/2_, respectively, while both peaks can be designated as W^6+^.^56,57^ Meanwhile, the peak that appears at 41.36 eV is designated as W 3p (Figure 3a). The XPS spectra of tungsten indicate that W is stabilized in VO_2_(M1) in a valent state of +6. It further reveals that the valence of W does not change before and after the reaction. Therefore, the charge contribution of W doping to VO_2_(M1) is 0. However, the V 2p spectrum of the W0.5 sample shows a change in the valence state of V after doping (Figure 3b).^58,59^ The peaks located at 516.4 and 524.5 eV can be assigned to V 2p_3/2_ and V 2p_1/2_, respectively. Meanwhile, the curve fitting can be categorized as peaks attributed to V^4+^ (located at 516.41 and 523.66 eV) and V^5+^ (517.93 and 525.05 eV). The ratio of the peak area of V^5+^ to that of V^4+^ is 3.44. This result suggests that parts of V lose electrons, resulting in an increase in the valence when W is doped with VO_2_(M1). In addition to the charge states of the metal elements, the bond information on N is discussed (Figure 3c).^60,61^ Adsorbed nitrogen (N_ab_, 401.82 eV) and N–H (399.82 eV) can be fingered in the N 1s spectra. However, the peak corresponding to the metal bonding (N–M) of N is not found (396 eV). The above phenomenon reveals that the N source present in the reaction is not involved in the reaction and is not doped into the VO_2_ structure.

The valence of Mo changed in Mo0.5 (Figure 3d).^58,59^ The Mo 3d spectrum showed a weak intensity. Mo 3d_5/2_ (230.19 and 232.49 eV) and Mo 3d_3/2_ (233.32 and 235.62 eV) are fingered after fitting. The peaks located at 230.19 and 233.32 eV can be attributed to Mo^4+^ and the peaks at 232.49 and 235.62 eV can be attributed to Mo^5+^. Meanwhile, the peak area shows Mo^4+^: Mo^5+^ = 1.34. The results of the Mo 3d spectral indicate that the valence of Mo changed before and after the reaction. Mo^6+^ joins the reaction to form 0.5 at. % Mo-VO_2_(M1), but Mo^5+^ and Mo^4+^ coexisted after doping. Therefore, the charge contribution of Mo doping to VO_2_(M1) lies between −1 and 0. In addition, the V 2p spectrum (Figure 3e) shows a 1.71 peak area ratio between V^5+^ and V^4+^.^58,59^ This result suggests that V also loses electrons, leading to an increase in the valence when Mo dopes with VO_2_(M1), and the increase of V valence may also cause a decrease in the valence of Mo. On the other hand, 0.5 at. % Mo doping causes a smaller amount of V to lose electrons and increase its valence than that of 0.5 at. % W doping. In addition, the N 1s spectra also show that there is no N doping into VO_2_ (Figure 3f).^60,61^ The XPS spectra of Cr0.5 have difficulty recognizing the charge state of Cr (Figure 3g). The intensity of the Cr 2p spectrum is too low, so it cannot be fitted (Figure S3). It is further indicated that the charge contribution of Cr doping to VO_2_ (M1) is also 0.^62^ However, the V 2p spectrum reveals a 1.70 peak area ratio between V^5+^ and V^4+^. This phenomenon suggests that the V valence is elevated after doping (Figure 3h).^53,54^ In addition, N is not doped into VO_2_(M1), which is similarly found from the N 1s spectra (Figure 3i).^60,61^ Thus, the XPS spectra reveal that the doping process elevates the valence of V. Meanwhile, N did not participate in the doping process of VO_2_(M1). In addition, the charge contributions of W0.5, Mo0.5, and Cr0.5 samples are 0, −1, ∼0, and 0, respectively, so that all samples make the Fermi level of VO_2_(M1) become lower (Figure 1).

**Figure 4:**
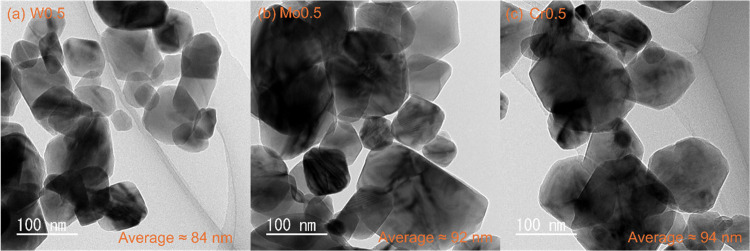
TEM images of (a) W0.5, (b) Mo0.5, and (c) Cr0.5 samples.

The TEM images provide feedback on the morphology information on W0.5, Mo0.5, and Cr0.5 samples. All three samples show a particle-like morphology. W0.5 shows the smallest particle size with an average particle size of about 84 nm (Figure 4a). When 0.5 at. % Mo is doped, the particle size of the sample slightly increased (Figure 4b). Its average particle size increased to 92 nm. 0.5 at. % Cr-doped VO_2_(M1) shows the largest crystal size with an average particle size of 94 nm (Figure 4c). The morphology information on W0.5, Mo0.5, and Cr0.5 samples indicates that the W0.5, Mo0.5, and Cr0.5 samples have the same morphology and similar particle size. At the same time, these three samples feedback similar specific surface areas (Table 1). Therefore, the effect of the specific surface area on the sensing behavior can be ignored when investigating gas sensing characteristics.

**Table 1:** Specific Surface Area of the Three Sensing Materials

sample	SSR (m^2^ g^–1^)^a^
W0.5	6.348
Mo0.5	6.209
Cr0.5	6.102

a Specific surface area.

### Sensing Behavior Modulation by W/Mo/Cr Doping

**Figure 5:**
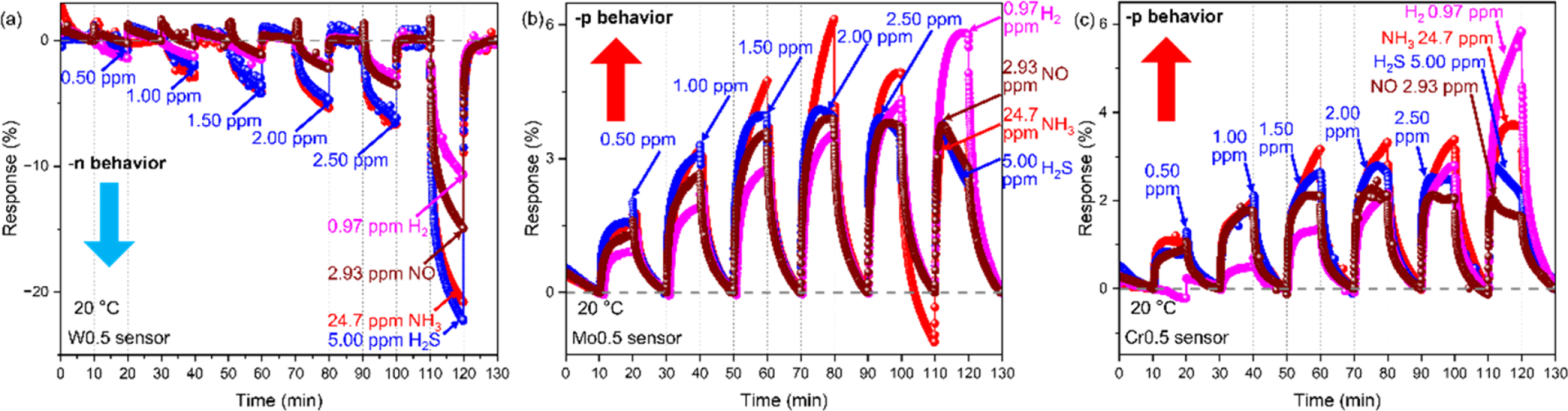
Gas sensing characteristics of (a) W0.5, (b) Mo0.5, and (c) Cr0.5 gas sensors at 20 °C.

Based on the thermionic emission theory (eq 4),^54,55^ element doping leads to changes in the Fermi level of the sensing material. The decreased Fermi level may further affect the formation of an inverse current between the sensing material and electrode by the Schottky junction. Therefore, element-doped sensing material has the potential to trigger anomalous sensing behavior. Hence, W0.5, Mo0.5, and Cr0.5 samples are chosen to evaluate their sensing behaviors to further clarify the effect of element doping on sensing behaviors. The sensing behavior of these three samples is investigated at 20 °C (Figure 5 and Table 2). As anticipated, the doping elements can modulate the sensing behavior of VO_2_(M1), facilitating a reverse from the n–p sensing behavior through doping at this temperature. NH_3_, H_2_S, H_2_, and NO are selected as target gases, respectively. Visually, the W0.5 sensor in the target gas shows a response due to a decrease in resistance (negative, -n). And it also shows an increase in the value in response to an increased concentration of target gas (Figure 5a). When NH_3_ is tested, the gas sensor feedbacks −0.68, −2.92, −4.10, −5.37, −6.60, and −20.37% show a response when correspondingly introduced 2.47, 4.94, 7.41, 9.88, 12.35, and 24.7 ppm of NH_3_ into the sensing system. W0.5 sensor shows a more pronounced response to H_2_S gas. The response of 0.50 ppm of H_2_S gas is −1.22%, while the response of 1.00, 1.50, 2.00, 2.50, and 5.00 ppm of H_2_S gas increases to −2.02, −4.24, −4.73, −6.58, and −22.27%, respectively. Meanwhile, 5.00 ppm of H_2_S shows the largest response in the investigated gas. Compared to the responses of NH_3_ and H_2_S, the W0.5 sensor feedback to H_2_ and NO is not as significant. Although the responses of these two gases still maintain the -n behavior and the response increases with increased gas concentration. However, the response is only −11.18% for 0.97 ppm of H_2_ and −14.93% for 2.93 ppm of NO. In addition, the W0.5 sensor is not significantly different for the four target gases on the response time (about 500 s; Figure S4a) and the recovery time (between 200 and 300 s; Figure S4a). Differently, the Mo0.5 sensor feedbacks a resistance-increase behavior (positive, -p) in all of the target gases, which corresponded to a response greater than 0 (Figure 5b). Mo doping resulted in a decrease in the response of VO_2_(M1) in the respective gases along with a poorer linearity of the response. Especially in 30–50% H_2_S or NO gas (corresponding to 1.50, 2.00, and 2.50 ppm for H_2_S or 0.88, 1.17, and 1.46 ppm for NO, respectively), the response of the gas maintains a similar value to the extent that it is difficult to distinguish the gas between the three concentrations. The Mo0.5 sensor maintains good linearity only for H_2_ gas and gives a maximum response (5.79%) at 0.97 ppm. This response is much higher than that of the other three gases at the maximum concentration (about 3.75%). Compared to the W0.5 sensor, the Mo0.5 sensor shows a faster response time for the four target gases (Figure S4b). The Mo0.5 sensor has a close response time for H_2_S, NO, and NH_3_ (below 200 s). Their response times are all below 200 s. However, it achieves a response time of about 440 s for H_2_. In addition, the recovery time of the Mo0.5 sensor for the four target gases is long and is greater than 500 s (Figure S4b). The sensing behaviors of W0.5 and Mo0.5 sensors reveal that changing the doping element can change the sensing ability of the sensing material for gases. The sensing behavior of these two sensors also shows good reproducibility at 20 °C (Figure S5a,b). Therefore, the sensing characteristics of W0.5 and Mo0.5 sensors are compared. The doping elements modulate the sensing behavior of VO_2_(M1), resulting in a -n behavior of VO_2_(M1) doped by W but -p behavior doped by Mo. It is further revealed that there may be two possible reasons for the modulation in the sensing behavior. One is for the reduction of the Fermi level by doping. The reduction of the Fermi level after gas absorption cannot cause the formation of Schottky barriers between the sensing material and the electrode when tungsten is doped, which in turn exhibits the -n behavior. However, molybdenum doping reduces the Fermi level deeply so that the Schottky barriers are formed between the sensing material and the electrode after gas absorption, which in turn leads to the elevation of the reverse current density and further contributes to the -p sensing behavior (Figure 1). Another reason is the different charge states of the dopant elements after doping. The different degrees of charge contribution between W^6+^ and Mo^5+/4+^ lead to differences in the charge-transfer mechanism during gas absorption (Figure 3a,d).

**Table 2:** Gas Sensing Behavior of W0.5, Mo0.5, and Cr0.5 Sensors

temperature (°C)	20	30	40	50	60
W0.5	-n	-n/-p^a^	-p	-p	-p
Mo0.5	-p	-p	-p	-p	-p
Cr0.5^b^	-p	-p	-p	-p	-p

a W0.5 feedbacks a concentration-induced inversion sensing behavior, which is shown in Table S2.

b Cr0.5 is unstable but mainly shows a -p behavior when increasing the operation temperature.

**Figure 6:**
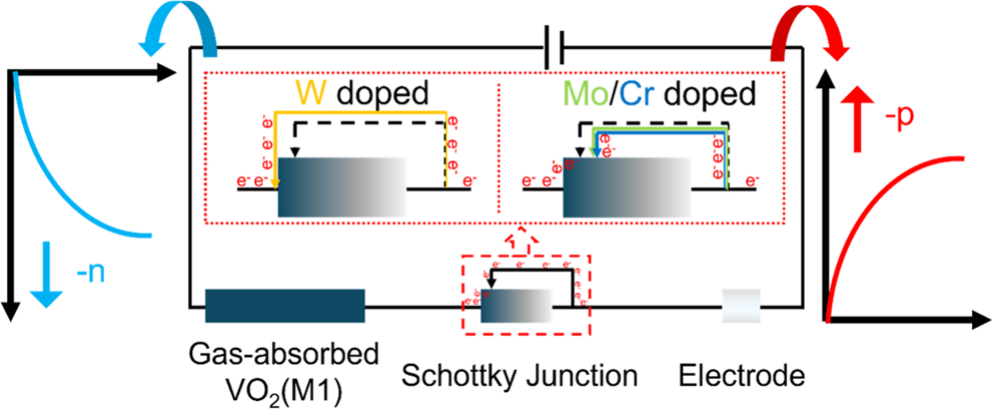
Mechanism diagram of VO_2_(M1) sensing behavior modulation by W/Mo/Cr doping.

However, the -p behavior of the Cr0.5 sensor for the four target gases (Figure 5c) negates the second hypothesis. The charge contribution of Cr doping for VO_2_(M1) is similar to that of W, but the sensing behavior of the Cr0.5 sensor is completely opposite to that of the W0.5 sensor. In addition, the Cr0.5 sensor still maintains good linearity to H_2_ as well as maximum sensing ability (5.84% for 0.97 ppm of H_2_, 3.52% for 24.7 ppm of NH_3_, 2.78% for 5.00 ppm of H_2_S, and 2.03% for 2.93 ppm of NO in response). Meanwhile, the Cr0.5 sensor shows rules of the response time and recovery time similar to those of the Mo0.5 sensor (Figure S4c). Its sensing characteristics also show good repeatability at 20 °C (Figure S5c). Therefore, the mechanism by which elements modulate the sensing behavior can be summarized as a process of the formation of a Schottky barrier driven by the Fermi level decreases. For easy understanding, the gas-absorbed sensing material after absorption, the Schottky junction, and the electrode are equated as three resistive elements in a circuit (Figure 6). The gas-absorbed sensing material and electrode are understood as constant-value resistors, while the Schottky junction created by the Schottky barrier is equated to a varistor. No Schottky junction is formed between VO_2_(M1) and the electrode when tungsten is doped, i.e., the varistor is shorted. Thus, the n-type VO_2_(M1) absorbs the reducing gases and manifests a -n behavior based on decreased resistance. Mo or Cr doping causes a Schottky junction to be formed between VO_2_(M1) and the electrode, which further increases reverse current density; i.e., a portion of the varistor is connected to the circuit. A portion of the reverse current generated by the Schottky barrier first offsets the original increase in the current of the sensor when gas is absorbed, i.e., offsets the decrease in resistance in sensing behavior. The remaining reverse current further causes the sensor to manifest -p behavior toward the reducing gas.

**Figure 7:**
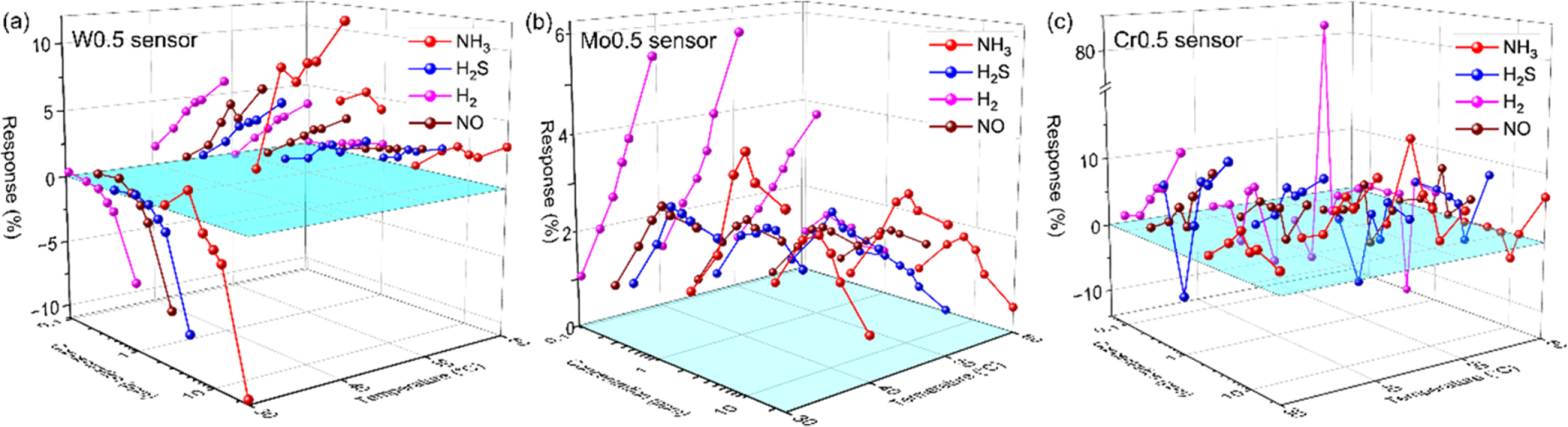
Gas sensing characteristics of (a) W0.5, (b) Mo0.5, and (c) Cr0.5 gas sensors at different temperatures.

More factors affecting the sensing behavior are expected to be revealed. Therefore, W0.5, Mo0.5, and Cr0.5 sensors are evaluated for sensing characteristics at operation temperatures of 30–60 °C (Figure 7, Tables 2 and S2). Surprisingly, the W0.5 sensor embodies diverse inversion behavior (Figure 7a). The response of the W0.5 sensor to the target gas is weaker at 30 °C than that at 20 °C. The sensing responses are −10.66, −8.06, −6.04, and −6.97% for 24.7 ppm of NH_3_, 5.00 ppm of H_2_S, 0.97 ppm of H_2_, and 2.93 ppm of NO, respectively. The sensing behavior of the W0.5 sensor at 30 °C seems to maintain -n. However, the lower concentration of the target gas shows unusual sensing behavior (Figure S6 and Table S2). The sensor is inverse to -p in 2.97 and 4.94 ppm of NH_3_. The sensing behavior inversed from -p to -n again when the NH_3_ concentration increased to 7.41 ppm (Figure S6a). As the gas concentration increases, the sensors show an increase (-p) to an increase (-n) in response. The low concentrations of H_2_S (0.50 and 1.00 ppm) also show inversion behavior of -p, and the sensing behavior also inverses again from -p to -n until the concentration increases to 1.50 ppm (Figure S6b). H_2_ also exhibits an inversion in the sensing behavior by the gas concentration. The sensing behavior is inverse to -p when the gas concentration is less than 0.29 ppm, and the gas concentration maintains -n consistent with that at 20 °C when the gas concentration is greater than 0.39 ppm (Figure S6c). The same inversion behavior is found in the NO sensing process (Figure S6d). 0.29, 0.59, 0.88, 1.17, and 1.46 ppm of NO correspond to 1, 1.15, 0.39, −0.28, and −1.33%, respectively. The above phenomenon indicates that the gas concentration is one of the factors affecting the sensing behavior. It is further revealed that the absorption amount of the gas molecule by the sensing material will influence the orientation of the resistance change of the sensing material.

The complete inversion sensing behavior of the W0.5 sensor occurs in the sensing characterization at 40 °C. The sensing behavior of the four gases is inversed from the initial -n to -p. Continuing to increase the operation temperature to 50 and 60 °C, the sensor consistently maintains -p. However, the response of the sensor to the four gases at 50 and 60 °C becomes weaker and weaker. At the same time, the response time and recovery time of the W0.5 sensor for all gases are significantly shortened and are characterized by fast response and quick recovery after the inversion sensing behavior occurs (Figure S4a). This is due to the change in the conductivity of the sensing material itself. As the operation temperature gets closer to its MST temperature (Figure S2a), the metallic phase becomes increasingly pronounced and the sensing material experiences an accelerated rate of electron transfer due to the increase in carrier concentration. This results in a nonsignificant change in the resistance of the sensing material after absorption of the gas. Although the W0.5 sensor gas sensor takes an inversion sensing behavior during the increase in operation temperature (30–60 °C), the sensing characteristics still remain -n in NH_3_ after cooling (Figure S7). It is indicated that the gas sensing characteristics of the W0.5 sensor maintain good repeatability. The inversion sensing behavior of the W0.5 sensor by temperature reveals that the operation temperature is another factor leading to anomalous sensing behavior, whereas the Mo0.5 sensor consistently maintains -p behavior and almost no change in response and recovery time for the target gas (Figures 7b and S4b). Meanwhile, the relationship between the response and operation temperature is consistent with that of the W0.5 sensor. The -p behavior, response time, and recovery time of the Cr0.5 sensor for the target gases remain unchanged (Figures 7c and S4c). However, the resistance of the Cr0.5 sensor becomes unstable when the operation temperature is adjusted to higher than 30 °C, which leads to an unstable response to gas. Therefore, it is difficult to construct a linear relationship between the gas concentration and the response. The temperature-sensing characteristics of the W0.5, Mo0.5, and Cr0.5 sensors also confirm the stability of the material after doping with these elements (Figure 1).

## Conclusions

This study explores the gas sensing characteristics of W/Mo/Cr-doped VO_2_(M1) to achieve the modulation of sensing behavior through elemental doping. Based on the relationship between the dopant amount and MST temperature, -n behavior is observed in W-doped VO_2_(M1), while -p behavior occurs in Mo/Cr-doped VO_2_(M1). This shift in sensing behavior is linked to the relationship between the Fermi level and the formation conditions of the Schottky junction. Additionally, factors such as the target gas concentration and operating temperature are also identified as influential in sensing behavior. This research provides a foundation for further advancements in the sensing theory.

## Supporting Information Available

Calibration of XRD patterns, DSC curves, XPS spectra of Cr 2p, response time and recovery time, repeatability at 20 °C, and time–response curves of the W0.5 sensor at 30 °C (PDF)
