# Supplementary material for: W/Mo/Cr Doping Modulates the Negative–Positive Inversion Gas Sensing Behavior of VO2(M1)
Source: ACS Sens. 2025 Jan 9;10(1):526–36. doi: 10.1021/acssensors.4c03006 (PMC11773573; doi:10.1021/acssensors.4c03006)
Supplement: Supplementary file 1 — se4c03006_si_001.pdf [file se4c03006_si_001.pdf]

Supporting Information

W/Mo/Cr doping Modulate the Negative-Positive Inversion Gas Sensing Behavior of VO_2(M1)

Lei Miao[1], Yibei Xue[1], Peng Song[1], Takuya Hasegawa[1], Ayahisa Okawa[1], Ryo Maezono[2], Tohru Sekino[3], and Shu Yin[1,4,*]

[1] Institute of Multidisciplinary Research for Advanced Materials (IMRAM), Tohoku University; Sendai 980-8577, Japan.

[2] School of Information Science, JAIST, Asahidai 1-1, Nomi, Ishikawa 923-1292, Japan

[3] SANKEN, Osaka University; Osaka 567-0047, Japan.

[4] Advanced Institute for Materials Research (WPI-AIMR), Tohoku University; Sendai 980-8577, Japan.

[*]Email: yin.shu.b5@tohoku.ac.jp

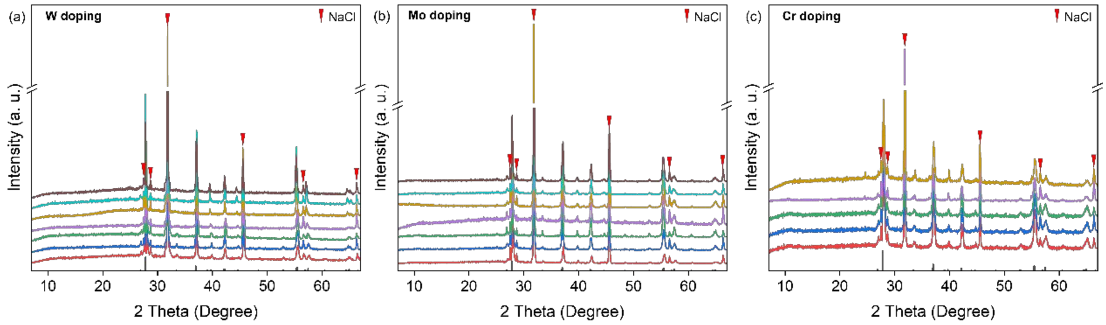

Figure S1 XRD patterns of the (a) W, (b) Mo, and (c) Cr doped VO_2(M1) samples which are calibrated by NaCl crystal. The XRD patterns correspond to that in Figure 2(a-c).

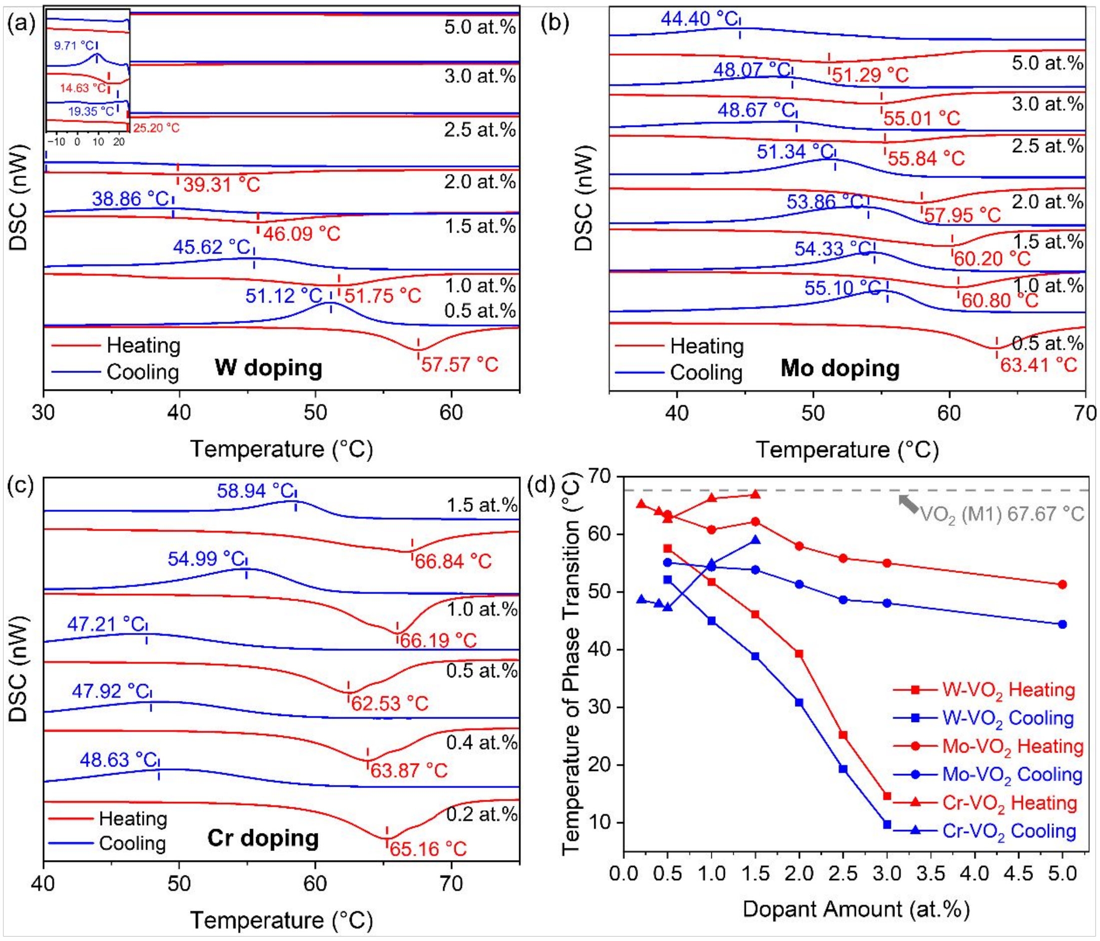

Figure S2 (a - c) DSC curves and (d) the relationship between dopant amount and the phase transition temperature of the (a) W, (b) Mo, (c) Cr doped VO_2(M1). The inset in (a) shows the DCS curves of 2.5, 3.0, and 5_{mol}%W-VO_2(M1) at -15 ~ 30 °C.

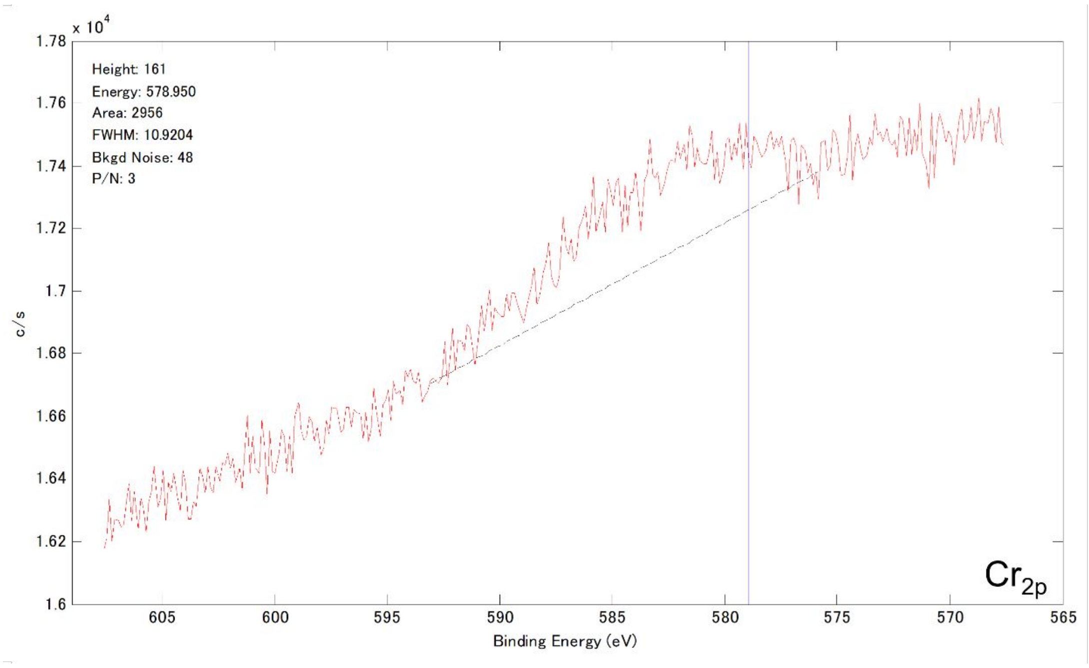

Figure S3 Cr 2p spectrum of Cr0.5 sample

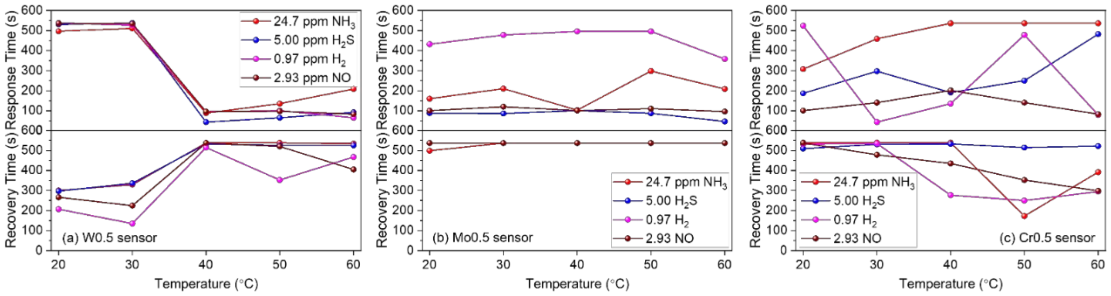

Figure S4 Response time and recovery time of (a) W0.5, (b) Mo0.5, and (c) Cr0.5 sensor, respectively.

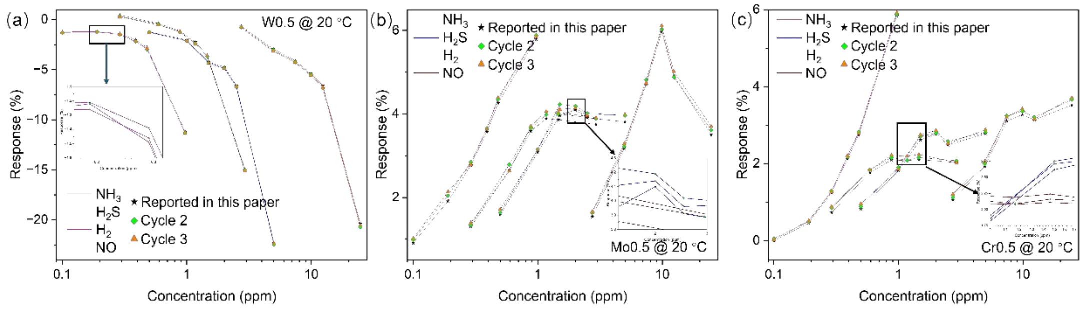

Figure S5 Repeatability of (a) W0.5, (b) Mo0.5, and (c) Cr0.5 sensor at 20 °C.

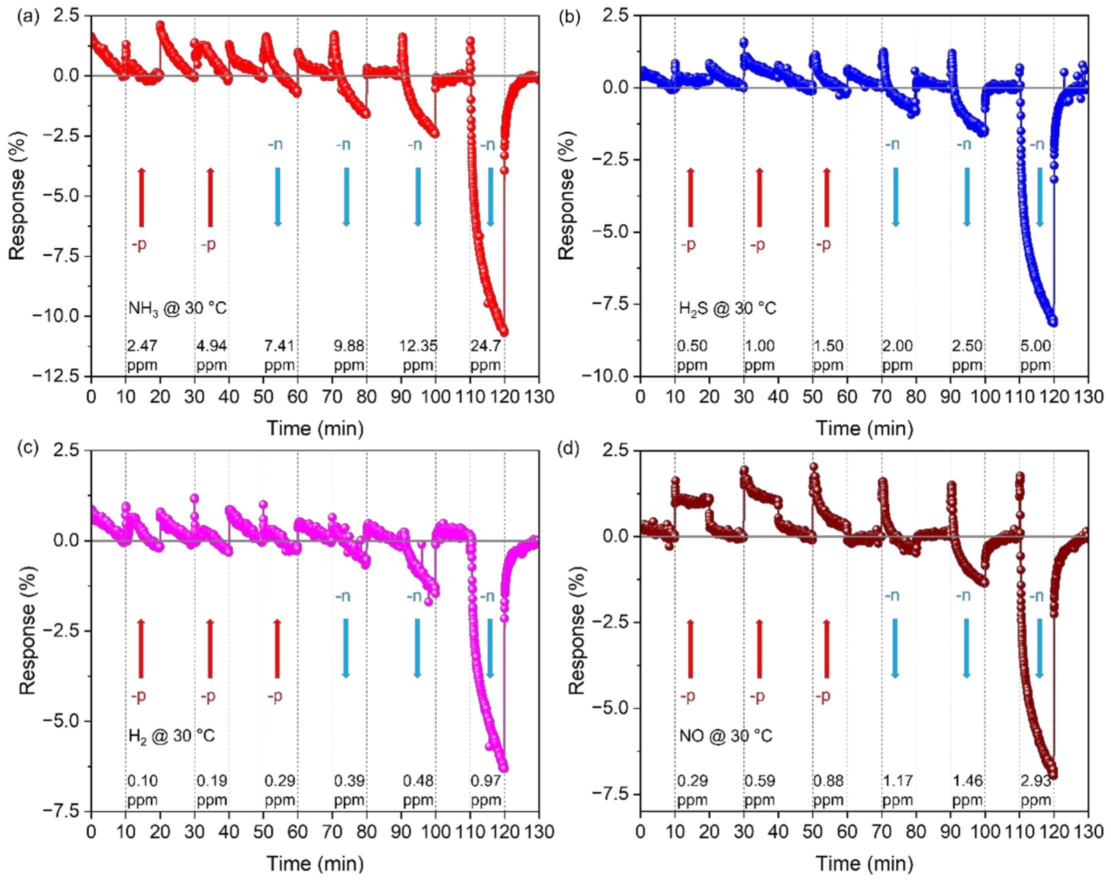

Figure S6 Gas sensing characteristics of W0.5 sensor to (a) NH_3, (b) H_2S, (c) H_2, (d) NO gas at 30 °C.

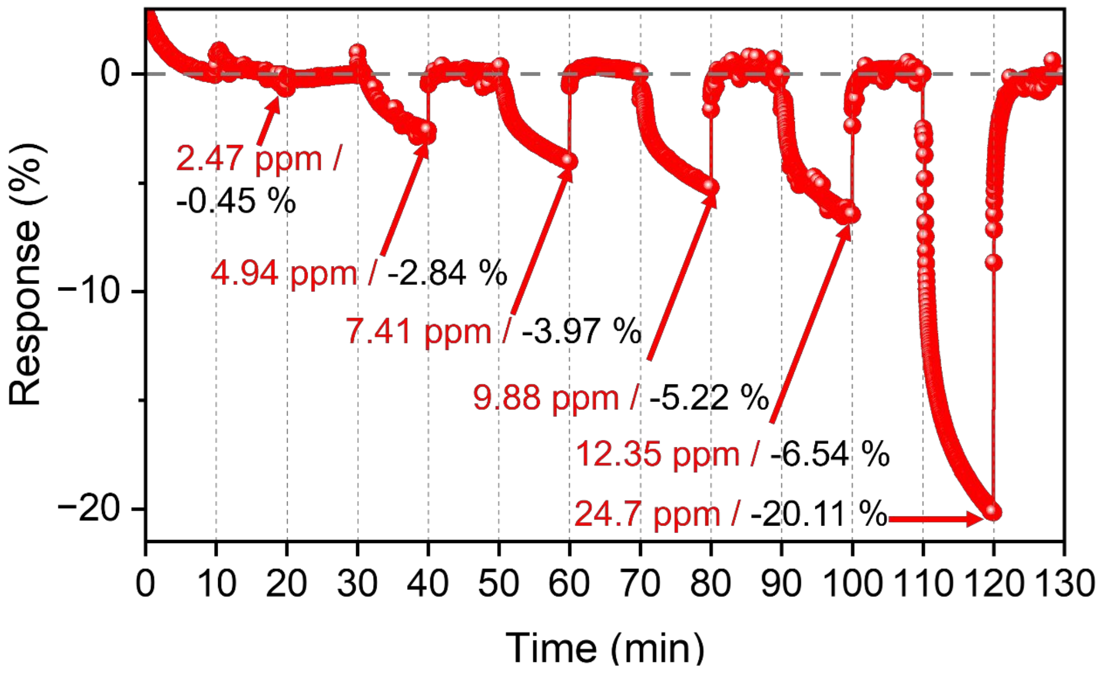

Figure S7 Gas sensing characteristics of the W0.5 sensor in NH_3 at 20 °C after cooling.

Table S1 Competing phases, standard energy of the reference phase (μ_0), and the deviation from the standard energy ($\Delta\mu_i$) for $V_{31/32}X_{1/32}O_2$ systems doped with different elements, sourced from the Material Project dataset. The chemical potential (μ_i) for each system is derived from the standard energy of the reference phase (μ_0) and the deviation ($\Delta\mu_i$) from this standard.

No.	*X*	Competing Phase	μ_0			$\Delta\mu_i$		
			V	O	X	V	O	X
1	Cr	$V_4Cr_2O_{13}$, VO_2, Cr_2O_3	-9.083906	-4.947961	-9.653047	-4.166104	-1.637361	-3.460427
2	Mo	V_3O_5, VO_2, MoO_2	-9.083906	-4.947961	-10.845650	-3.153396	-2.143715	-1.777235
3	W	VO_2, V_2WO_6, WO_3	-9.083906	-4.947961	-12.958130	-3.828798	-1.806015	-3.345398
4	Ti	VO_2, TiV_2O_7, TiO_2	-9.083906	-4.947961	-7.895492	-4.380132	-1.530348	-7.444219
5	Sc	VO_2, $ScVO_4$, V_2O_5	-9.083906	-4.947961	-6.332469	-5.157830	-1.141499	-8.527396
6	Mn	VO_2, MnV_2O_6, V_2O_5	-9.083906	-4.947961	-9.162015	-5.157830	-1.141499	-3.837644
7	Fe	VO_2, $V_4Fe_2O_{13}$, Fe_2O_3	-9.083906	-4.947961	-8.470021	-4.266714	-1.587056	-1.887144
8	Zr	VO_2, ZrV_2O_7, ZrO_2	-9.083906	-4.947961	-8.547701	-3.740786	-1.850021	-7.740812
9	Nb	V_3O_5, VO_2, Nb_2O_5	-9.083906	-4.947961	-10.101305	-3.153396	-2.143715	-5.260430
10	Hf	VO_2, HfV_2O_7, HfO_2	-9.083906	-4.947961	-9.957189	-3.595891	-1.922468	-8.214497
11	Ta	$TaVO_4$, VO_2, $TaVO_5$	-9.083906	-4.947961	-11.85778	-3.466025	-1.987401	-6.830142

Table S2 Gas sensing behavior of W0.5 sensor at 30 °C

Flow Rate (sccm)*	180:20	160:40	140:60	120:80	100:100	0:200
NH_3 (24.7 ppm)	-p	-p	-n	-n	-n	-n
H_2S (5.00 ppm)	-p	-p	-p	-n	-n	-n
H_2 (0.97 ppm)	-p	-p	-p	-n	-n	-n
NO (2.93 ppm)	-p	-p	-p	-n	-n	-n

* The flow rate of base gas and target gas (base gas: target gas) when W0.5 test at different concentrations.
